# Effects of emulsifiers on lipid metabolism and performance of yellow-feathered broilers

**DOI:** 10.1186/s12917-024-04095-8

**Published:** 2024-06-07

**Authors:** Yuxuan Wang, Dewei Zeng, Limin Wei, Jingshen Chen, Hongyi Li, Lijun Wen, Guangming Huang, Zhenqing Dai, Junyi Luo, Jiajie Sun, Qianyun Xi, Yongliang Zhang, Ting Chen

**Affiliations:** 1 College of Animal Science, Guangdong Province Key Laboratory of Animal Nutritional Regulation, National Engineering Research Center for Breeding Swine Industry, State Key Laboratory of Livestock and Poultry Breeding, South China Agricultural University, Guangzhou, Guangdong 510642 China; 2 Hainan Key Laboratory of Tropical Animal Breeding and Epidemic Research, Institute of Animal Husbandry and Veterinary Research, Hainan Academy of Agricultural Sciences, Haikou, Hainan 571100 China; 3 Yingdong College of Biology and Agriculture, Shaoguan University, Shaoguan, Guangdong 512005 China; 4 Guangdong Hainachuan Biotechnology Co., LTD, Guangzhou, Guangdong 528515 China

**Keywords:** Yellow-feathered broiler, Growth performance, Emulsifying agent, Lipid metabolism

## Abstract

**Background:**

Reducing production costs while producing high-quality livestock and poultry products is an ongoing concern in the livestock industry. The addition of oil to livestock and poultry diets can enhance feed palatability and improve growth performance. Emulsifiers can be used as potential feed supplements to improve dietary energy utilization and maintain the efficient productivity of broilers. Therefore, further investigation is warranted to evaluate whether dietary emulsifier supplementation can improve the efficiency of fat utilization in the diet of yellow-feathered broilers. In the present study, the effects of adding emulsifier to the diet on lipid metabolism and the performance of yellow-feathered broilers were tested. A total of 240 yellow-feasted broilers (21-day-old) were randomly divided into 4 groups (6 replicates per group, 10 broilers per replicate, half male and half female within each replicate). The groups were as follows: the control group (fed with basal diet), the group fed with basal diet supplemented with 500 mg/kg emulsifier, the group fed with a reduced oil diet (reduced by 1%) supplemented with 500 mg/kg emulsifier, and the group fed with a reduced oil diet supplemented with 500 mg/kg emulsifier. The trial lasted for 42 days, during which the average daily feed intake, average daily gain, and feed-to-gain ratio were measured. Additionally, the expression levels of lipid metabolism-related genes in the liver, abdominal fat and each intestinal segment were assessed.

**Results:**

The results showed that compared with the basal diet group, (1) The average daily gain of the basal diet + 500 mg/kg emulsifier group significantly increased (*P* < 0.05), and the half-even-chamber rate was significantly increased (*P* < 0.05); (2) The mRNA expression levels of *Cd36*, *Dgat2*, *Apob*, *Fatp4*, *Fabp2*, and *Mttp* in the small intestine were significantly increased (*P* < 0.05). (3) Furthermore, liver TG content significantly decreased (*P* < 0.05), and the mRNA expression level of *Fasn* in liver was significantly decreased (*P* < 0.05), while the expression of *Apob*, *Lpl*, *Cpt-1*, and *Pparα* significantly increased (*P* < 0.05). (4) The mRNA expression levels of *Lpl* and *Fatp4* in adipose tissue were significantly increased (*P* < 0.05), while the expression of *Atgl* was significantly decreased (*P* < 0.05). (5) Compared with the reduced oil diet group, the half-evading rate and abdominal fat rate of broilers in the reduced oil diet + 500 mg/kg emulsifier group were significantly increased (*P* < 0.05), and the serum level of LDL-C increased significantly (*P* < 0.05)0.6) The mRNA expression levels of *Cd36*, *Fatp4*, *Dgat2*, *Apob*, and *Mttp* in the small intestine were significantly increased (*P* < 0.05). 7) The mRNA expression levels of *Fasn* and *Acc* were significantly decreased in the liver (*P* < 0.05), while the mRNA expression levels of *Lpin1*, *Dgat2*, *Apob*, *Lpl*, *Cpt-1*, and *Pparα* were significantly increased (*P* < 0.05).

**Conclusions:**

These results suggest that dietary emulsifier can enhance the fat utilization efficiency of broilers by increasing the small intestinal fatty acid uptake capacity, inhibiting hepatic fatty acid synthesis and promoting hepatic TG synthesis and transport capacity. This study provides valuable insights for the potential use of emulsifier supplementation to improve the performance of broiler chickens.

## Background

With the continuous improvement of genetic strains, the production cycle of commercial broilers has become shorter, leading to increased nutritional demands and a higher need for feed energy. Lipids constitute the main energy source for animals, among all the nutrients, lipids contribute the highest caloric value [1]. In order to meet the high-energy demand of modern commercial broilers, it is often necessary to supplement feed with oil to enhance its energy content [2, 3]. Different from mammals, the main source of fat in chickens is de novo synthesis of liver fat [4]. Previous studies have shown that appropriately increasing the oil content in the diet of yellow-feathered broilers can not only fulfill the high-energy demand of broilers, but also supply essential fatty acids for growth, promote the absorption, transportation, and utilization of fat-soluble vitamins in the diet, and enhance palatability, thereby improving broilers productivity [5–7]. Soybean oil is predominantly used as the added oil in the diet, but its price is high and is expected to further increase in the future [6, 8, 9]. Consequently, reducing production costs while ensuring the production of high-quality livestock and poultry products remains a perennial concern in the livestock industry. Improving dietary energy utilization can effectively reduce production costs [10, 11]. Emulsifier can be used as a potential feed supplement to enhance the energy utilization rate and sustain the high productivity of broilers, potentially replacing a portion of the oil in broiler diets [12, 13]. Additionally, due to the incomplete development of gastrointestinal function and insufficient bile acid secretion in adult broilers, they are unable to fully emulsify dietary fat and effectively utilize fat in the feed, leading to feed wastage and issues such as broiler diarrhea [14, 15]. Therefore, adding emulsifier to broiler feed can facilitate more comprehensive emulsification of oil intake, increase the contact area between chyme and intestinal digestive enzymes, so as to promote the utilization of broiler diet lipids [7, 16]. With the advancement of the broiler breeding industry, the application of emulsifiers has garnered increasing attention as a means to improve the utilization rate of feed oil and reduce production costs.

The aim of this study is to enhance the utilization efficiency of lipids in yellow-feathered broiler diets by adding emulsifiers. It seeks to investigate the impact of emulsifiers on the growth performance and lipid metabolism of yellow-feathered broilers, assess the practical application effects of emulsifiers, improve the utilization rate of lipids in the diet to optimize broiler feed, reduce production costs, and enhance economic efficiency. The study aims to provide a theoretical foundation for the application of emulsifiers in the broiler production industry to enhance the utilization efficiency of dietary oil.

## Results

### Performance of production

Compared with the basal diet group, the basal diet + 500 mg/kg emulsifier group showed a significant increase in Average daily gain (ADG) from 43 to 63 days of age (*P* < 0.05). Additionally, compared with the oil-reduced diet group, the Feed-Gain ratio (F/G) of broilers aged 43–63 days in the oil-reduced diet + 500 mg/kg emulsifier group showed a trend of decrease (*P* < 0.10) (Table 1). These results suggest that the addition of emulsifiers may improve oil utilization and thus reduce feeding costs.

**Table 1:** Effects of emulsifier on growth performance of yellow-feathered broilers(*n* = 6)

Items	Times	basal diet	basal diet + 500 mg/kg emulsifier	reduced oil diet	reduced oil diet + 500 mg/kg emulsifier
ADFI/g	21–42 d	48.56 ± 1.83	48.53 ± 4.14	49.38 ± 2.69	48.92 ± 0.86
	43–63 d	73.73 ± 3.02	72.94 ± 3.70	73.90 ± 2.67	72.94 ± 2.40
	21–63 d	61.14 ± 2.03	60.94 ± 3.68	61.64 ± 2.57	60.93 ± 1.13
ADG/g	21–42 d	19.51 ± 0.98	19.56 ± 2.99	20.50 ± 1.52	19.92 ± 0.94
	43–63 d	24.88 ± 1.02^b^	26.20 ± 0.88^a^	26.05 ± 0.52^ab^	25.66 ± 0.61^ab^
	21–63 d	22.68 ± 0.76	22.68 ± 1.72	23.23 ± 1.09	22.98 ± 0.60
F/G	21–42 d	2.49 ± 0.11	2.51 ± 0.20	2.41 ± 0.06	2.46 ± 0.10
	43–63 d	2.91 ± 0.06	2.90 ± 0.09	2.90 ± 0.08	2.82 ± 0.01
	21–63 d	2.70 ± 0.04	2.68 ± 0.07	2.65 ± 0.08	2.65 ± 0.04

Test results are expressed as Mean ± SD. In the same row, values with no letter or the same letter superscripts mean no significant difference (*P*>0.05), while with different small letter superscripts mean significant difference (*P*<0.05). Abbreviations: ADFI Average daily feed intake, ADG Average daily gain, F/G Feed-Gain ratio.

### Performance of slaughter

Compared with the basal diet group, the half-eviscerated rate of broilers in the basal diet + 500 mg/kg emulsifier group was significantly increased (*P* < 0.05), and the eviscerated rate showed an upward trend (*P* < 0.10). Additionally compared with the oil-reduced diet group, the half-eviscerated rate and abdominal fat rate of broilers in the oil-reduced diet + 500 mg/kg emulsifier group were significantly increased (*P* < 0.05). Furthermore, in comparison with the basal diet group, the half-eviscerated rate of broilers in the oil reduction diet + 500 mg/kg emulsifier group was significantly increased (*P* < 0.05) (Table 2). These results suggest that the addition of emulsifier can improve the performance of yellow-feathered broilers.

**Table 2:** Effects of emulsifier on carcass performance yellow-feathered broilers

Items	basal diet	basal diet + 500 mg/kg emulsifier	reduced oil diet	reduced oil diet + 500 mg/kg emulsifier
Live weight (g)	1184.56 ± 42.71	1186.15 ± 80.05	1208.24 ± 52.31	1198.59 ± 21.26
Dressed percentage (%)	90.25 ± 0.93	90.55 ± 0.97	90.58 ± 1.44	90.35 ± 1.31
Half eviscerated percentage (%)	81.61 ± 1.93^a^	83.77 ± 0.93^b^	82.09 ± 1.73^a^	83.51 ± 1.39^b^
Eviscerated percentage (%)	65.88 ± 1.66	66.94 ± 1.17	66.29 ± 1.58	66.83 ± 0.89
Breast muscle percentage (%)	13.92 ± 1.53^ab^	13.07 ± 1.36^b^	14.44 ± 0.82^a^	14.09 ± 0.72^ab^
Leg muscle percentage (%)	21.17 ± 1.53	20.26 ± 2.18	21.36 ± 1.56	20.73 ± 1.49
Abdominal fat percentage (%)	2.02 ± 0.68^ab^	2.33 ± 0.71^ab^	1.85 ± 0.48^a^	2.56 ± 0.75^b^

Test results are expressed as Mean ± SD. In the same row, values with no letter or the same letter superscripts mean no significant difference (*P* > 0.05), while with different small letter superscripts mean significant difference (*P* < 0.05).

### Effect of emulsifier on lipid metabolism in yellow-feathered broilers

#### Serum biochemical indicators

Compared with oil-reduced diet group, the serum Low density liptein cholesterol (LDL-C) content of broilers in the oil-reduced diet + 500 mg/kg emulsifier group was significantly increased (*P* < 0.05). In addition to that, compared with the basal diet group, the serum LDL-C content of broilers in the oil reduction diet + 500 mg/kg emulsifier group was significantly increased (*P* < 0.05) (Table 3). These results suggest that the addition of emulsifier improves the transport of liver-synthesized lipids to other tissues in yellow-feathered broilers.

**Table 3:** Effects of emulsifier on serum biochemical indexes of yellow-feathered broilers

Items	basal diet	basal diet + 500 mg/kg emulsifier	reduced oil diet	reduced oil diet + 500 mg/kg emulsifier
TG(mmol/L)	0.38 ± 0.02	0.36 ± 0.02	0.38 ± 0.02	0.35 ± 0.03
T-CHO(mmol/L)	3.33 ± 0.11	3.19 ± 0.09	3.31 ± 0.12	3.45 ± 0.15
HDL-C(mmol/L)	3.59 ± 0.20	3.27 ± 0.16	3.47 ± 0.14	3.35 ± 0.22
LDL-C(mmol/L)	1.39 ± 0.07^a^	1.45 ± 0.08^a^	1.50 ± 0.07^a^	1.79 ± 0.14^b^

Test results are expressed as Mean ± SEM. In the same row, values with no letter or the same letter superscripts mean no significant difference (*P*>0.05), while with different small letter superscripts mean significant difference (*P*<0.05). Abbreviations: TG Triglyceride, T-CHO Total cholesterol, HDL-C High density liptein cholesterol, LDL-C Low density liptein cholesterol.

#### mRNA expression levels of lipid metabolism genes in the small intestinal

**Fig. 1:**
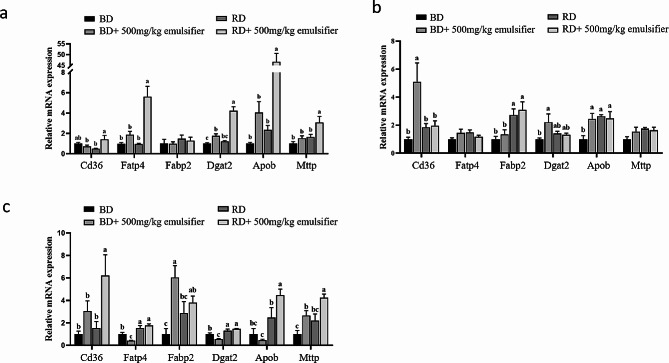
mRNA expression level of lipid metabolism genes in small intestinal. (**a**) Duodenum. (**b**) Jejunum. (**c**) Ileum. *Abbreviations*: *Cd36* Fatty acid translocase, *Fatp4* Fatty acid transport protein 4, *Fabp2* Fatty acid binding protein 2, *Dgat2* Diacylglycerol acyltransferase 2, *Apob* Apolipoprotein B, *Mtttp* Microsomal triglyceride transfer protein, BD Basal die, RD Reduced oil diet

In order to determine the effects of dietary emulsifiers on lipid metabolism in the small intestine of broilers, the mRNA expression levels of Fatty acid translocase (*Cd36*), Fatty acid transport protein 4 (*Fatp4*), Fatty acid binding protein 2 (*Fabp2*), TG and Diacylglycerol acyltransferase 2 (*Dgat2*), Apolipoprotein B (*Apob*), and Microsomal triglyceride transfer protein (*Mttp*) in duodenum, jejunum and ileum segments of broilers were measured. *Cd36*, *Fatp4*, and *Fabp2* play a crucial role as a carrier for fatty acid uptake and transport in the body [17, 18]. *Dgat2* can promote the formation of lipid droplets in the intestine or adipose tissue [19]. *Apob* and *Mttp* are mainly involved in the efficient assembly and secretion of lipoproteins [20, 21]. Compared with the basal diet, the mRNA expression level of *Dgat2* in the duodenum of the basal diet + 500 mg/kg emulsifier group was significantly increased (*P* < 0.05) (Fig. 1a). Additionally, the mRNA expression levels of *Cd36*, *Dgat2*, and *Apob* in jejunum was significantly increased (*P* < 0.05) (Fig. 1a). Moreover, the mRNA expression levels of *Fabp2* and *Mttp* in the ileum was significantly increased (*P* < 0.05) (Fig. 1b), while the mRNA expression levels of *Fatp4* and *Dgat2* in the ileum was significantly decreased (*P* < 0.05) (Fig. 1c). Compared with the oil-reduced diet, the mRNA expression levels of *Cd36*, *Fatp4*, *Dgat2*, *Apob*, and *Mttp* in the duodenum of the oil-reduced diet + 500 mg/kg emulsifier group were significantly increased (*P* < 0.05) (Fig. 1a). Furthermore, the mRNA expression levels of *Cd36*, *Apob* and *Mttp* in ileum were significantly increased (*P* < 0.05) (Fig. 1c). The above results suggest that the addition of emulsifier may promote oil utilization by improving the absorption and transport capacity of oil in the intestine.

#### Effects of emulsifier on liver fat content and mRNA expression levels of lipid metabolism genes in the liver

**Fig. 2:**
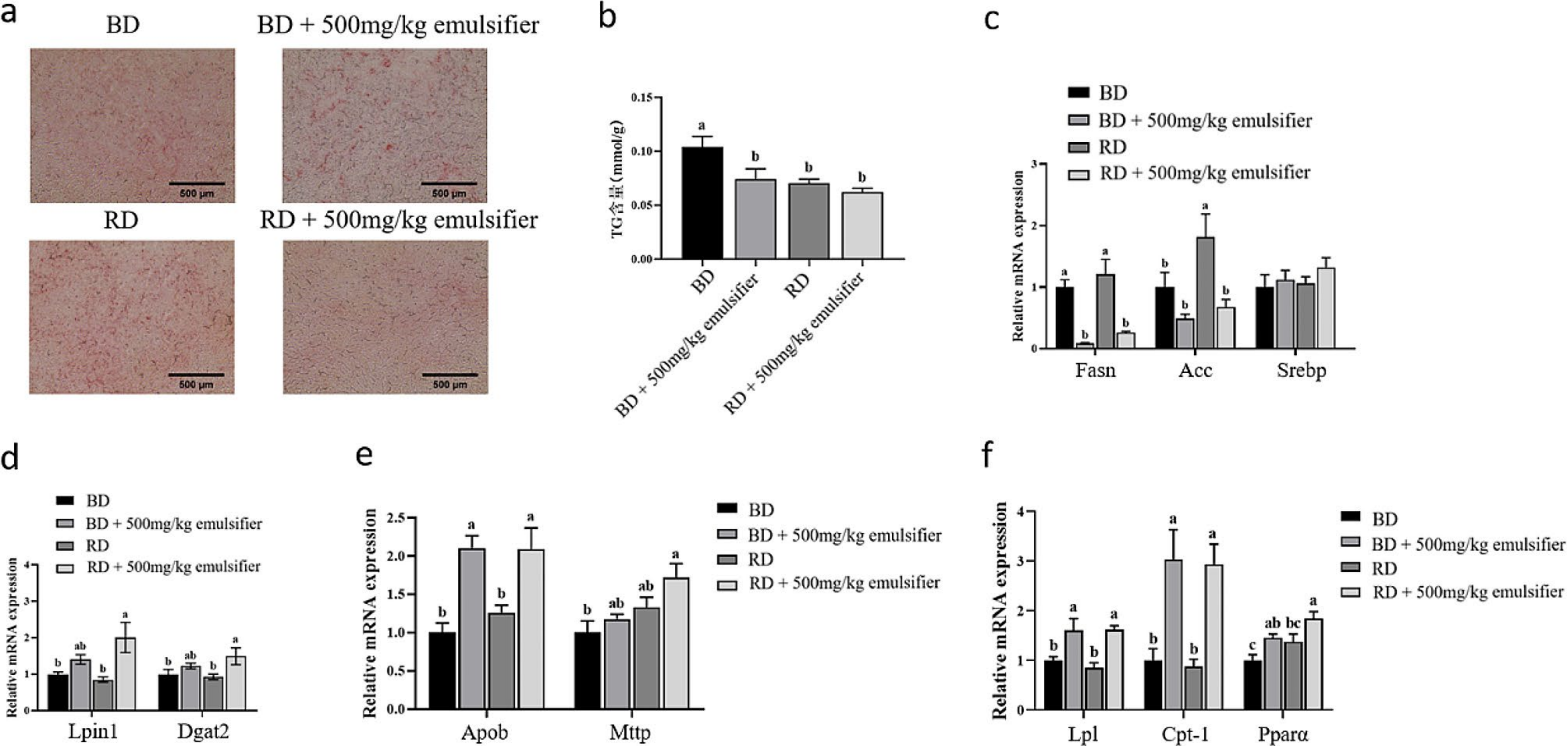
Effect of emulsifier on liver fat content in yellow-feathered broilers (*n* = 6). (**a**) The liver was stained with oil red O. (**b**) Liver TG content. (**c**) Hepatic fatty acid synthesis related gene expression. (**d**) Hepatic TG synthesis related gene expression. (**e**) Expression of genes involved in liver lipid transport. (**f**) Expression of genes involved in hepatic lipolysis. *Abbreviations*: *Fasn* Fatty acid synthase, *Acc* Acetyl-CoA carboxylase, *Srebp* Sterol regulatory element-binding protein, *Lpin1* Lipid phosphate phosphohydrolase, *Dgat2* Diacylglycerol acyltransferase 2, *Apob* Apolipoprotein B, *Mttp* Microsomal triglyceride transfer protein, *Lpl* Lipoprotein lipase, *Cpt-1* Carnitine palmitoyltransferase 1, *Pparα* Peroxisome proliferator-activated receptor alpha

Compared with the basal diet group, the liver TG content of the basal diet + 500 mg/kg emulsifier group decreased significantly (*P* < 0.05) (Fig. 2ab), and the mRNA expression level of Fatty acid synthase (*Fasn*), a gene involved in fatty acid synthesis, was significantly reduced in the liver (*P* < 0.05) (Fig. 2c), Additionally, the expression levels of *ApoB*, Lipoprotein lipase (*Lpl*), Carnitine palmitoyltransferase 1 (*Cpt-1*), and Peroxisome proliferator-activated receptor alpha (*Pparα*) were significantly increased (*P* < 0.05) (Fig. 2ef). There was no significant difference in liver TG content between the oil-reduced diet + 500 mg/kg emulsifier group and the oil-reduced diet group (*P* > 0.05) (Fig. 2ab). The mRNA expression levels of *Fasn* and Acetyl-CoA carboxylase (*Acc*) in the liver were significantly decreased (*P* < 0.05) (Fig. 2c), and the mRNA expression levels of Lipid phosphate phosphohydrolase (*Lpin1*), *Dgta2*, *Apob*, *Lpl*, *Cpt-1*, and *Pparα* were significantly increased (*P* < 0.05) (Fig. 2def). These results suggest that dietary emulsifiers can inhibit hepatic fatty acid synthesis and increase hepatic TG synthesis, transport and decomposition, so that more TG synthesized by the liver can be transported to various tissues of broilers.

#### mRNA expression levels of lipid metabolism genes in the adipose tissue

**Fig. 3:**
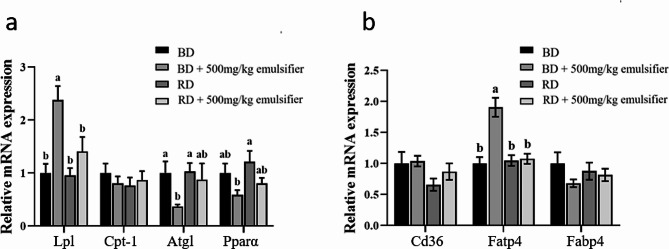
mRNA expression level of lipid metabolism genes in adipose tissue (*n* = 6). (**a**) Expression of genes involved in abdominal lipolysis. (**b**) Expression of genes involved in abdominal fatty acid transport. *Abbreviations*: *Lpl* Lipoprotein lipase, *Cpt-1* Carnitine palmitoyltransferase 1, *Atgl* Adipose triglyceride lipase, *Pparα* Peroxisome proliferator-activated receptor alpha, *Cd36* Fatty acid translocase, *Fatp4* Fatty acid transport protein 4, *Fabp4* Fatty acid binding protein 4

Compared with the basal diet, the mRNA expression level of lipolysis related gene *Lpl* was significantly increased, while the expression of Adipose triglyceride lipase (*Atgl*) was significantly decreased (Fig. 3a), Additionally, the mRNA expression level of the fat transport-related gene *Fatp4* was significantly increased in the basal diet + 500 mg/kg emulsifier group (Fig. 3b). There were no significant differences in the remaining lipolysis and transport-related genes. It was speculated that dietary emulsifier could improve fatty acid transport capacity in abdominal adipose tissue of broilers.

## Discussion

### Effect of emulsifier on growth performance of yellow-feathered broilers

Several studies have shown that dietary emulsifier supplementation significantly increased body weight, average daily gain, average daily feed intake and reduced feed weight of broiler [22–25]. Furthermore, the addition of lecithin to the broiler diet has been shown to significantly improves the digestibility of feed fat, leading to increased weight gain of broilers and improving feed conversion rate [26–28]. The results of this experiment align with these findings, as dietary emulsifier supplementation significantly increased and promoted the ADG of yellow-feathered broilers aged 43 to 63 days, consistent with the results of the aforementioned studies.

### Effect of emulsifier on slaughter performance of yellow-feathered broilers

Slaughter performance is an important indicator that directly reflects the growth performance of animals, thereby affecting the economic benefits of livestock and poultry breeding [29, 30]. In this experiment, we found that dietary emulsifiers effectively improved the slaughter performance of broilers, increasing the half-eviscerated rate and abdominal fat rate of yellow-feathered broilers, while achieving partial replacement of oil. Similar studies have also demonstrated that feeding broilers with emulsifier supplementation diets increased the fat percentage on day 35 [31]. Additionally, feeding lysophospholipid at the inclusion levels of 0.10% and 0.15% in reduced energy diets led to a superior increase in body weight and body weight gain of birds [32]. Furthermore, broilers fed lysolecithin gained more body weight when consuming the lysolecithin at either 25–50 g/kg at 21 days of age and 50 g/kg at 35 days of age, with feed efficiency improving with 50 g/kg diet [33]. These results indicate that the addition of emulsifier to the diet can improve the performance of broiler chickens. However, the requirement of emulsifier varies with the age of chickens, which is the direction of future research. In addition, this study only investigated broilers from 21 days to 63 days of age, and the effects on performance of broilers at other days of age need to be further studied.

These results align with the current study, suggesting that emulsifier-supplemented broilers met their nutrient needs for their productivity when emulsifier was added to reduced nutrient diets. Contradictory studies on the effect of emulsifiers on abdominal fat have also reported, which showed that adding emulsifier reduced abdominal fat percentage in 24 and 28-day-old broilers [10, 11]. The different results on abdominal fat percentage may be attributed to the different emulsifier and emulsifier level in the diet, as well as different sampling periods. We speculate that further study is needed to understand the role of emulsifiers in regulating lipid metabolism of broilers at different feeding stages.

### Effect of emulsifier on lipid metabolism in yellow-feathered broilers

#### Effect of emulsifier on serum biochemistry of yellow-feathered broilers

The contents of TG, T-CHO, HDL-C, and LDL-C in serum can directly reflect the body’s lipid metabolism, especially the fatty acid metabolism in the liver and adipose tissue [34–36]. In the current study, it showed that de-oiled lecithin diet did not affect the concentrations of T-CHO and TG, but enhanced the concentrations of serum HDL-C and LDL-C [37]. When emulsifier was added to the feed of meat ducks, it reduced TG in serum and promoted the digestion and absorption of fat for the ducks [38]. The results of this study indicate that dietary emulsifier can increase the serum LDL-C level of broilers. And LDL-C can regulate the endogenous lipid transport capacity of yellow-feathered broilers [39, 40]. LDL-C can transport lipids synthesized by the liver to the outside of the liver [41, 42]. These findings suggest that dietary emulsifier has a certain ability to regulate the lipid metabolism of broilers, but the specific action pathway remains to be studied.

#### Effect of emulsifier on mRNA related to lipid metabolism in the small intestine of yellow-feastered broilers

Studies have shown that lysolecithins are added to poultry diets to promote the intestinal absorption of nutrients, particularly of dietary fats [12]. To further determine the pathway of action of emulsifiers in regulating lipid metabolism in broilers, the present study examined the expression of genes related to lipid metabolism in the small intestine of broilers. *Cd36* is very important for fatty acid uptake and lipid metabolism, serving as a crucial carrier for fatty acid uptake, transport, and lipid deposition in the body [17, 43]. *Fatp4*, the only member of the fatty acid transporter family found in the intestine, plays a key role in the absorption of long-chain fatty acids [18, 44]. *Fabp2* expresses the most *Fabp* in the intestine, facilitating the transport of lipids from the intestinal lumen to enterocytes [45]. *Dgat2* plays a significant role in the absorption of fat in the small intestine [19], it induces TG synthesis and promote lipid droplet formation in intestinal or adipose tissue [19, 46, 47]. *Apob* and *Mttp* are essential for the efficient assembly and secretion of lipoproteins [20, 21], they are required for chylomicron production in the small intestine [48, 49]. In the present study, the mRNA expression levels of *Cd36*, *Fabp2*, *Dgat2*, *Apob*, and *Mttp* in the small intestine were increased by adding emulsifiers to either the basal diet or the oil-reduced diet, suggesting that dietary emulsifiers may enhance the expression levels of genes related to fatty acid transport and chylomicum composition in the small intestine of broilers. thereby promoting the ability of broilers to take up fat from food.

#### Effect of emulsifier on liver lipid metabolism of yellow-feathered broilers

To further explore the effect of dietary emulsifiers on the lipid metabolism of broilers, the expression levels of liver lipid metabolism-related genes were measured. Different from mammals, the main source of fat in avian species is de novo synthesis of liver fat [4, 50, 51]. *Acc* and *Fasn* encode key enzymes for de novo fatty acid synthesis [52, 53]. *Srebps* are hepatic transcription factors that regulate the expression of genes required for fatty acid synthesis, thereby controlling fatty acid uptake, biosynthesis, and cholesterol biosynthesis [54, 55]. *Lpin* and *Gdat2* are mainly involved in TG synthesis, and increased expression of *Lpin* and *Gdat2* results in increased TG synthesis [56, 57]. *Apob* and *Mttp* are mainly involved in liver lipid transport. The increased expression of *Apob* and *Mttp* can promote lipid transport from the liver to other sites. VLDL particles are formed in the endoplasmic reticulum, where *Apob* is lipidated in a process catalyzed by the enzyme *Mttp* [58]. The nascent VLDL particle is then transferred to the Golgi apparatus, and during this process, the particle is further lipidated until a mature VLDL particle is formed [59]. Consequently, *Apob* and *Mttp* are key components in hepatic VLDL secretion. Lpl is a key enzyme in lipolysis, it can hydrolyze VLDL and TG in chylomicrons to glycerol and fatty acids for use by various tissues [60, 61]. Cpt-1 catalyzes the formation of fatty acyl carnitine and is the rate-limiting enzyme in fatty acid oxidation [62, 63]. *Pparα*, mainly expressed in the liver and abdominal fat, enhances fatty acid β oxidation and reducing lipid deposition [64, 65]. The results of this study showed that dietary emulsifier supplementation reduced TG content and the expression levels of the fatty acid synthesis related gene (*Fasn*), while increasing hepatic TG synthesis (*Lpin*, *Gdat2*), transport (*Apob*), and breakdown (*Lpl*, *Cpt-1*, *Pparα*) gene expression levels in broilers. Therefore, it is speculated that dietary emulsifier can inhibit fatty acid synthesis ability of liver, increase TG synthesis, transport, and decomposition ability of liver, and cause more TG synthesized by the liver to transported to various tissues of broilers.

#### Effect of emulsifier on lipid metabolism in adipose tissue of yellow-feathered broilers

To further confirm the action pathway of dietary emulsifiers on lipid metabolism in broilers, the expression levels of genes related to lipolysis and fatty acid transport in abdominal fat were measured. Abdominal fat is the main site of fat deposition in broilers [66]. *Lpl* is a gene related to lipolysis [60]. Studies have shown that severe hypertriglyceridemia, reduced high density lipoprotein, and neonatal death in *Lpl* knockout mice. Mild hypertriglyceridemia with impaired very low density lipoprotein clearance in heterozygotes [67]. And *Fatp4* is a gene related to fat transport [68]. Studies have shown that adipocyte-specific inactivation of *Fatp4* causes adipose hypertrophy [69]. The results of this study showed that the dietary addition of 500 mg/kg emulsifier significantly increased the mRNA expression levels of *Lpl* and *Fatp4* in abdominal fat. Similar studies have demonstrated that dietary emulsifier supplementation reduces the relative content of abdominal fat [10]. Therefore, it is speculated that dietary emulsifier will improve the fatty acid transport capacity of abdominal adipose tissue in broilers.

## Conclusions

In conclusion, dietary emulsifier supplementation significantly promoted ADG in yellow-feathered broilers aged 43 to 63 days, and increased the percentage of half evisceration rate and abdominal fat of yellow-feathered broilers. This effect may be related to improving the performance of broilers by increasing the expression of chylomicrons-related genes in the small intestine, promoting the ability of the small intestine to take up fatty acids from food, inhibiting fatty acid synthesis in the liver, and promoting TG synthesis and transport in the liver. The appropriate addition of emulsifiers to the diet can improve the utilization rate of oil, thereby reducing the use of oil and reducing the feeding cost. However, the specific amount of oil reduction remains to be further studied.

## Methods

### Experimental design and diets

The emulsifiers used in this study were provided from Guangdong Hainachuan Biotechnology Co., Ltd. (Guangdong, China). It primarily consisted of lecithin and lysolecithin, with a content of approximately 25%. The experimental diet was provided by Foshan Huayang Animal Nutrition Products Co., Ltd. (Guangdong, China) and was formulated in accordance with the nutritional requirements of broiler chickens in China (NY/T 33-2004). And the dose is 500 mg/kg feed. The dose used in the current study was selected based on the study of Ahmed A et al [70] and V. Bontempo et al [71]. The composition and nutrient levels of diets at different stages were see in Table 4.

The experiment was completed in the chicken farm of Guangdong Qingnong New Agricultural Technology Co., Ltd. (Guangdong, China). A total of 240 yellow-feathered broilers (half male and half female) aged 21 days were randomly divided into 4 groups (6 replicates per group, 10 broilers per replicate, half male and half female within each replicate): the basal diet group, oil-reduced diet group, oil-reduced diet + 500 mg/kg emulsifier group, and basal diet + 500 mg/kg emulsifier group. Each group had 6 replicates, with each replicate consisting of 10 broilers. The broilers were raised in two stages: from 21 to 42 days old and from 43 to 63 days of age. The duration of the experiment was 42 days. Throughout the experiment, food and water were provided ad libitum, and lighting was maintained throughout the day.

**Table 4:** Composition and nutrient levels of experimental diets (air-dry basis, %)

Items	Content			
	21 to 42 days		43 to 63 days	
	basal diet	reduced oil diet	basal diet	reduced oil diet
Ingredients				
Corn	62.6	63.6	61.9	62.9
Soybean meal	30	30	27.7	27.7
Soybean oil	3.8	2.8	6.8	5.8
Limestone	1.11	1.11	1.31	1.31
CaHPO_4_	1	1	0.8	0.8
NaCl	0.25	0.25	0.25	0.25
Antiseptic	0.1	0.1	0.1	0.1
premix^1)^	0.12	0.12	0.12	0.12
L-Lys•HCl (98.5%)	0.32	0.32	0.33	0.33
DL-Met (99%)	0.27	0.27	0.28	0.28
L-Thr (97.5%)	0.08	0.08	0.07	0.07
Choline chloride (50%)	0.1	0.1	0.1	0.1
Antioxidant	0.1	0.1	0.1	0.1
Phytase	0.02	0.02	0.02	0.02
β-Mannan	0.03	0.03	0.02	0.02
Dysenteriae essential oil	0.02	0.02	0.02	0.02
Sodium humate	0.05	0.05	0.05	0.05
Monensin	0.03	0.03	0.03	0.03
Total	100	100	100	100
Nutrient levels^2)^				
Metabolizable energy(MJ/Kg)	12.78	12.55	13.47	13.27
Crude protein	18.183	18.339	17.153	17.229
Crude fat	5.892	4.903	8.853	7.884
Crude fiber	2.772	2.778	2.626	2.642
Ca	0.740	0.737	0.728	0.728
Total phosphorus	0.497	0.497	0.449	0.451
Non-phytate phosphorus	0.258	0.258	0.221	0.222
Lys	1.170	1.168	1.117	1.119
Thr	0.828	1.023	0.773	0.776
Met	0.543	0.542	0.538	0.539
Met + Cys	0.859	0.859	0.837	0.841

1. The premix provided the following per kg of diets: VA 8000 IU, VD_3_ 2000 IU, VK_3_ 4 mg, VE 20 mg, VB_1_ 2 mg, VB_2_ 6 mg, VB_6_ 4 mg, VB_12_ 0.02 mg, nicotinamide 4 mg, folic acid 1 mg, D-pantothenic acid 12 mg, biotin 0.12 mg, Cu (as copper sulfate) 1–25 mg, Fe (as ferrous sulfate) 5-180 mg, Mn (as manganese sulfate) 8-150 mg, Zn (as zinc sulfate) 15–120 mg, I (as Calcium iodate) 0.1-4 mg, Se (as sodium selenite) 0.1–0.5 mg, Co (as cobalt sulfate) 0.1-1 mg.
2. Nutrient levels were calculated values.

### Rearing management and sample collection

The experiment was completed in the chicken farm of Guangdong Qingnongxin Agricultural Science and Technology Co., Ltd.(Guangdong, China). The number of deaths and the health status of the chickens were recorded. When the chickens reached the age of 63 days, the chickens were euthanized by cervical dislocation after blood samples collection, and samples of the small intestine, liver, and fat were collected and and immediately frozen in liquid nitrogen and stored at − 80 °C for RNA analyses.

### Performance of growth

The average daily feed intake (ADFI) was calculated as the average amount of feed consumed daily during the growth trial. ADG for each chicken was expressed as the ratio of body weight to age in days of rearing. After a 12-hour fast at 42 and 63 days of age, the chickens were weighed.

### Performance of slaughter

When the chickens reached 63 days of age, two chickens (one male and one female) were randomly selected from each replicate. The live weight of the chickens was weighed, after which they were slaughtered and plucked. The carcass weight, half-eviscerated weight, eviscerated weight, pectoral muscle weight, leg muscle weight, and abdominal fat weight were recorded.

### Serum biochemical indicators

After fasted for 12 h, two chickens (one male and one female) were randomly selected from each replicate, and blood was collected from the wing vein. The serum was then separated and stored at -20 °C. Serum triglyceride (TG, A110-1-1), total cholesterol (T-CHO, A111-1-1), high density lipoprotein cholesterol (HDL-C, A112-1-1), and low density lipoprotein cholesterol (LDL-C, A113-1-1) were measured using kits provided by Nanjing Jiancheng Bioengineering Institute (Nanjing, China). The relevant indicators were measured, and the data were analyzed according to the instructions of the kit.

### mRNA expression level of lipid metabolism genes

Total RNA was extracted from the small intestine, liver, and fat using Trizol reagent (Invitrogen, 153 Carlsbad, CA, USA) according to the manufacturer’s protocol, and a reverse transcription kit (EZB A0010CG) was used for reverse transcription. RT-qPCR was performed according to the instructions of RealStar Fast SYBR (A301-10) to determine the expression of mRNA. The sequences of the primers utilized in RT-qPCR assays are presented in Table 5, and the relative mRNA expression of the above target genes was calculated by the 2-ΔΔCt method using GAPDH as the reference gene.

**Table 5:** Primer sequences for RT-qPCR

Gene	Forward Primer	Reverse Primer	Size/bp	NCBI Reference Sequence
*Acc*	GCTGGGTTGAGCGACTAATGA	GAAACTGGCAAAGGACTGACG	171	NM_205505.2
*Fasn*	TGCTATGCTTGCCAACAGGA	ACTGTCCGTGACGAATTGCT	128	NM_205155.4
*Srebp-1c*	TACCGCTCATCCATCAACGAC	TTCCTCAGGATCGCCGACTT	92	NM_204126.3
*Lpin1*	TTGGTGCTGATGGCGTCTA	CGAAGGCAAATCTCTGGTT	212	XM_015276089.4
*Dgat2*	GTGAAAACCCACAACCTGCT	CTATGCTGTCACGGTTCACG	232	XM_040661934.2
*Mtttp*	CAAGAACCGGATAGCCATGT	AGGAGAGCCAACTCTGTCCA	175	NM_001109784.3
*Apob*	ATGTTCCACAGGACCTACGC	TGCAGTGCATCAATGACAGA	214	NM_001044633.2
*Cd36*	GCGATTTGGTTAATGGCACT	TCTCCAACATCAATCGGTGA	196	NM_001030731.1
*Fabp4*	GCCTGACAAAATGTGCGACC	TTCCTGGTAGCAAACCCCAC	105	NM_204290.2
*Fabp2*	GGAGCCCACGATAATCTGAA	TTTCCTTCCAGGTTCCAAGA	170	NM_001007923.2
*Fatp4*	AAAGGGGATGCTGCCTATCT	CCCATACACAACCACGTCTG	177	XM_015279553.4
*Lpl*	TTGGTGACCTGCTTATGCTA	TGCTGCCTCTTCTCCTTTAC	185	NM_205282.2
*Atgl*	CCTTTGGACTCCGCTTGGAA	GGACCCAGGAACCTCTTTCG	242	NM_001113291.2
*Cpt-1*	GCCCCTCTAGCTGGCTTATT	AGGGAGTACCGCATTGTGAC	205	NM_001012898.1
*Pparα*	GGTGGACACTGAAAACCAGC	CTGGATGCTGGTGAAAGGGT	228	NM_001001464.1
*Gapdh*	AGTCGGAGTCAACGGATTTG	ACAGTGCCCTTGAAGTGTCC	165	NM_204305.2

### Data analysis

Feeding production data were expressed as mean ± Standard Deviation (SD). Other data were expressed as mean ± standard error of mean (SEM) and subjected to GraphPad Prism. Comparisons between two groups were assessed by the student t test. One-way ANOVA was used to compare three or four groups. *P* < 0.05 was considered as statistical significance.

## Data Availability

The original contributions presented in the study are included in the article. Further inquiries may be directed to the corresponding author.
